# Differentiating ‘pure’ and comorbid self-identified burnout: Diagnostic and management implications

**DOI:** 10.1177/10398562241236119

**Published:** 2024-02-29

**Authors:** Gabriela Tavella, Gordon Parker

**Affiliations:** Discipline of Psychiatry and Mental Health, School of Clinical Medicine, 7800 The University of New South Wales, Sydney, NSW, Australia

**Keywords:** burnout, depression, psychiatric history, mixture modelling

## Abstract

**Objective:**

A previous study identified categorically differing scores on the Sydney Burnout Measure (SBM) between individuals with self-identified burnout with (*n* = 354) or without (*n* = 188) a history of mental illness. The current study examined whether the SBM’s validity held in both scenarios.

**Method:**

The factorial structure and scores on the SBM measure were compared between the two groups.

**Results:**

Similar underlying symptom constructs were identified. The group with a mental illness history had higher general factor scores, suggesting more severe burnout. The group without such a history (and thus a ‘purer’ burnout state) had higher scores on the empathy loss factor, suggesting its greater specificity to burnout than to other psychological states.

**Conclusions:**

Burnout appears to be experienced similarly by those with and without a mental illness history as measured by the SBM.

Burnout is most commonly defined as a syndrome comprised of emotional exhaustion, depersonalisation/cynicism and reduced professional accomplishment,^1,2^ a definition adopted in ICD-11. Our previous studies^3–4^ led to the development of the Syndey Burnout Measure (SBM), with broader constructs of exhaustion, cognitive impairment, loss of empathy, insularity, impaired work performance and an unsettled mood (including feel drained, sad and frustrated).

We have argued^3,4^ that, due to burnout’s overlap with many other psychological states, especially depression,^5^ most burnout measures have low specificity, whereby an individual with another psychological condition may score highly on the measure and generate a ‘false positive’ burnout diagnosis. If burnout is a categorical ‘pure’ state, then scores on burnout measures should show a unimodal distribution reflecting a single construct varying in severity. In our previous study,^6^ 622 Australian participants with self-diagnosed burnout recruited through online advertisements completed a questionnaire examining features of their burnout (including symptoms included in the SBM), management strategies trialed, and whether they had been previously diagnosed with (i) depression or (ii) any other mental health condition. Refined mixture modelling analyses of those data indicated a two-class (i.e. bimodal) distribution of SBM scores. Those in the higher scoring class were more likely to have reported having a previous and/or ongoing mental health condition (depression or some other condition) in addition to their burnout. Considering the contentious overlap between burnout and depression, we pursued whether excluding those nominating a prior depression diagnosis led to a unimodal distribution of SBM scores. The distribution remained bimodal, suggesting that the two-class solution was not merely a result of those in the higher scoring class conflating burnout and depressive symptoms. The distribution only became unimodal after excluding participants nominating any previous mental health diagnosis (i.e. not just depression), indicating that the presence of any such diagnoses resulted in a less ‘pure’ burnout syndrome.

The current study examined whether the factorial structure underlying the SBM (a bifactor solution comprising five specific factors – exhaustion, cognitive impairment, empathy loss, insularity/social withdrawal and impaired work performance – and a general factor encompassing those domains plus unsettled mood) was maintained in those reporting a history of mental illness in addition to experiencing burnout. Equivalent factor structures would argue for an additive model (i.e. individuals truly having burnout superimposed upon other mental illness symptoms) as against those with a history of mental illness generating a ‘false positive’ burnout diagnosis.

## Method

The study was approved by the University of New South Wales Human Research Ethics Committee and the 622 participants^6^ provided written informed consent. In determining if the factor structure underlying the SBM burnout model was comparable for both those with (MI+) and without (MI-) a previous and/or ongoing mental illness, we calculated and compared ‘model fit’ statistics (comparative fit [CFI], Tucker–Lewis [TLI], and root mean square error approximation [RMSEA] indices) for the SBM 34-item bifactor model in each group. CFI and TLI indices >0.90 and RMSEA indices between 0.08 and 0.05 indicate adequate model fit.^7,8^ As a more formal test of model equivalence across the two groups, we examined for measurement invariance, which would indicate that a model has psychometric equivalence across groups.^9^ Scores on each SBM factor were then compared between the groups.

Group differences in reporting each of 34 SBM items were examined by comparing the odds of responding ‘moderately’ or ‘distinctly’ versus ‘not at all’ or ‘slightly’ for each item using the SPSS GENLOG function,^10^ adjusted to control the false discovery rate (FDR).^11^

## Results

Table 1 displays fit indices of the SBM solution for the whole sample as well as for the MI+ (*n* = 354) and MI- (*n* = 188) groups. Model fit was adequate for both groups, indicating a similar factor model structure. Partial measurement (i.e. scalar) invariance was also established, indicating that the underlying symptom constructs were similar in both groups, and allowing examination as to whether the factor means differed for the two groups. As reported in Table 2, the MI + group had a higher mean factor score on the general factor and a lower mean factor score on the empathy loss factor.

**Table 1.** SBM model fit indices for the whole sample and groups of participants based on mental illness history status

	Model fit indices		
	RMSEA	CFI	TLI
Whole sample (*n =* 542)	0.06	0.94	0.93
MI- (*n* = 188)	0.06	0.93	0.93
MI+ (*n =* 354)	0.06	0.95	0.94

CFI = comparative fit index; MI- = no mental illness history. MI+ = mental illness history; RMSEA = root mean square error approximation; SBM = Sydney Burnout Measure; TLI = Tucker–Lewis index.

**Table 2.** Factor score means for those with and those without a previous mental illness history

	Mean^a^		SE	*p* value^b^
SBM factor	MI−	MI+		
General factor	0.00	0.39	0.11	.00*
Cognitive dysfunction	0.00	0.16	0.12	.18
Empathy loss	0.00	−0.23	0.12	.04*
Exhaustion	0.00	−0.33	0.29	.26
Reduced work performance	0.00	0.00	0.13	.99
Social withdrawal	0.00	0.22	0.12	.08

MI- = no mental illness history; MI+ = mental illness history; SE = standard error; SBM = Syndey Burnout Measure.

^a^Factor score means were fixed to zero for the MI- group in the standardised solution of the partial scalar invariance model.

^b^Tests were deemed significant if *p* < .05 and are indicated with *.

The percentage of each MI+ and MI- group rating each of the SBM items as ‘moderately’ or ‘distinctly’ present was quantified. Only four generated significant odds ratios (ORs), with the MI + group having greater odds of reporting ‘I feel sad, empty and hopeless’, ‘I withdraw from family and friends’, ‘I stop looking forward to spending time with friends and family’ and ‘I am less productive at work’ than the MI-group, with the first three seemingly capturing depressive items.

As displayed in Table 3, no significant demographic differences were quantified between the groups other than ethnicity and education levels. Those in the MI + group were more likely to report having (i) stopped working due to their burnout, (ii) consulted a general practitioner or mental health professional, (iii) taken an antidepressant or (iv) any other medication (psychotropic or otherwise), and (v) presented to hospital because of burnout symptoms.

**Table 3.** Comparison of demographic and other variables between the MI- and MI + groups

	MI- group (*n* = 188)	MI+group (*n* = 354)	Test statistic^a^	*p* value^b^
Mean age	42.0 (*SD* = 11.5)	41.4 (*SD* = 11.0)	0.6	0.55
Gender	78.2% female	79.0% female	0.1	0.82
Ethnicity^c^	63.3% Australian	79.1% Australian	15.8	0.00*
Education level	84.6% university degree	76.6% university degree	4.8	0.03*
Employment status	89.4% employed full- or part-time	83.6% employed full- or part-time	3.3	0.07
Most frequently nominated occupation	11.2% education professional (e.g. teacher and school principal)	10.7% education professional (e.g. teacher and school principal)	0.0	0.88
Stopped working due to burnout	23.9%	52.3%	40.3	0.00*
Consulted a general practitioner to manage burnout symptoms	28.2%	62.7%	58.5	0.00*
Consulted a mental health professional to manage burnout symptoms	29.8%	68.9%	76.1	0.00*
Took an antidepressant medication to manage burnout symptoms	3.7%	55.1%	138.6	0.00*
Took some other medication to manage burnout symptoms	8.0%	19.8%	12.9	0.00*
Went to hospital to manage burnout symptoms	0.5%	7.6%	12.6	0.00*

MI- = no mental illness history; MI+ = mental illness history.

^a^The test statistic for age was a Student’s *t* value (*df* = 540), all other test statistics were *χ*^*2*^. values (*df* = 1).

^b^Tests were deemed significant if *p* < .05 and are indicated with *.

^c^All participants were recruited from Australia but some nominated ethnicities other than “Australian” that best described them.

## Discussion

Our earlier paper^6^ considered whether those with self-identified burnout might be validly identifying such a condition. Refined analyses of SBM measure scores demonstrated a bimodal pattern, which appeared to capture individuals with ‘pure’ burnout and those with a previous and/or current mental health problem.

Current analyses indicated that the two groups shared the same underlying set of burnout constructs, but their expression varied in severity across the two groups, with higher SBM scores in the MI+ group presumably reflecting comorbid psychological or other conditions and consequently generating higher SBM scores, supporting an ‘additive’ model. MI+ group members were also more likely to report having ceased work, sought medical assistance, taken an antidepressant or other medication and/or presented at hospital because of their burnout. Such differences could reflect MI + members having experienced a more severe burnout syndrome and/or be a consequence of their previous and/or ongoing psychological states.

Previous research has highlighted substantial interdependence between burnout and depression in particular.^12^ Our finding of higher scores on several depressive items in the MI+ group could indicate that the bimodal distribution in SBM scores simply reflected those with burnout and those with depression, but would likely have prevented model fit being demonstrated across the two groups. Our demonstration of model fit argues here for an additive model, with both groups experiencing burnout but with its greater overall severity in the MI + group reflecting the impact of previous and/or ongoing psychological problems increasing total SBM scores.

In the overall sample, 37% of the sample reported having trialled an antidepressant drug to treat their ‘burnout’, which could be interpreted as suggesting that antidepressant drugs are helpful for treating burnout. In the present study, however, the rates of antidepressant trialling were distinctly different in the MI+ (55%) and MI- (3%) groups, suggesting that any antidepressant was more likely to have been prescribed for their previous and/or ongoing concomitant depressive symptoms. Such a finding illustrates the importance of defining those with a ‘pure’ burnout syndrome in evaluating causal and management strategies for ‘burnout’ specifically.

Factor scores for the two groups indicated that those in the MI- group (and having a ‘purer’ burnout syndrome) returned significantly higher empathy loss scores, thus suggesting its possible greater relevance to burnout than to other psychological syndromes. In support of that interpretation, our earlier analysis^4^ quantified that those with burnout had a significantly higher empathy loss scale score than a group of participants with clinically diagnosed depression.

### Clinical implications and further research

The results of the current study reflect a reality that psychiatrists are likely to face when assessing and managing burnout – some individuals will experience ‘pure’ burnout while others will experience the syndrome accompanied by comorbid psychological conditions. That many of those in the MI + group had trialled antidepressants to treat their ‘burnout’ might encourage the view that antidepressants are an appropriate treatment option for burnout, despite the medications more likely treating co-occurring depressive symptoms and there being little empirical evidence of antidepressant effectiveness for burnout. Whether an individual has ‘pure’ versus comorbid burnout thus appears influential in diagnosis and treatment decisions, and these influences should be further examined in future studies. Along this line, the SBM’s specificity should be further explored in samples of individuals with a range of mental conditions (in addition to burnout) to determine its diagnostic utility and whether differential cut-offs indicative of ‘pure’ versus comorbid burnout should be derived. Other factors, such as specific biomarkers of burnout compared to other conditions, especially depression, may need to be considered, and future research is needed to determine whether such specific markers exist.

### Limitations

The study was limited by the use of self-report. In future studies, participants’ reports of burnout should be complemented with diagnostic impressions from a clinician (in light of there currently being no validated diagnostic interview for burnout), with a focus on also identifying any other preceding or co-occurring mental health conditions. In addition, while we specifically asked about depression history, for those nominating another mental health diagnosis we did not ask them to specify the condition, nor did we assess whether such conditions were ongoing or in remission at the time of participants’ burnout.

## Conclusions

While the burnout label resonates with many and thus many individuals readily self-diagnose as being burnt out, clinicians should expect that those who present with a self-diagnosis of burnout may have such a condition alone or one that is accompanied by another psychological state (or even a physical condition). Indeed, our current analyses indicated that those who self-identify as experiencing burnout likely comprise two groups – those with the ‘pure’ syndrome and those with the syndrome accompanied by comorbid psychological conditions. Our finding of similar factorial constructs in both study groups supports the validity and utility of the SBM in capturing burnout in both such clinical presentations.

## Data Availability

The data generated during and/or analysed during the current study are not publicly available nor are they available on request due to consent for data sharing not sought when data was collected.
